# Development and validation of prognostic nomogram in ependymoma: A retrospective analysis of the SEER database

**DOI:** 10.1002/cam4.4151

**Published:** 2021-08-03

**Authors:** Zetian Jia, Yaqi Yan, Jiuxin Wang, He Yang, Haihua Zhan, Qian Chen, Yawei He, Changyu Huang, Yuhua Hu

**Affiliations:** ^1^ Department of Neurosurgery The Second Hospital of Hebei Medical University Shijiazhuang People’s Republic of China; ^2^ Department of Cardiology The First Hospital of Handan of Hebei Province Handan People’s Republic of China; ^3^ Department of Gastrointestinal Surgery Xianyang First People's Hospital Xianyang People’s Republic of China

**Keywords:** ependymoma, nomogram, prognosis, SEER

## Abstract

**Background:**

The prognostic factors for survival in patients with ependymoma (EPN) remain controversial. The aim of this study was to establish a prognostic model for 5‐ and 10‐year survival probability nomograms for patients with EPN.

**Methods:**

Clinical data from the Surveillance, Epidemiology, and End Results (SEER) database were used for patients diagnosed with ependymoma between 2000 and 2018 and were randomized 7:3 into a development set and a validation set. Factors significantly associated with prognosis were screened out using the least absolute shrinkage and selection operator (LASSO) regression. The calibration chart and consistency index (C‐index) are used to evaluate the discrimination and consistency of the prediction model. Decision curve analysis (DCA) was used to further evaluate the established model. Finally, prognostic factors selected by LASSO regression were evaluated using Kaplan–Meier (KM) survival curves.

**Results:**

A total of 3820 patients were included in the prognostic model. Seven survival predictors were obtained by LASSO regression screening, including age, gender, morphology, location, size, laterality, and resection. The prognostic model of the nomogram showed moderate discriminative ability in the development group and the validation group, with a C‐index of 0.642 and 0.615, respectively. In the development set and validation set survival curves, the prognosis index of high risk was less effective than low risk (*p* < 0.001).

**Conclusions:**

Our nomograms may play an important role in predicting 5 and 10‐year outcomes for patients with ependymoma. This will help assist clinicians in personalized medicine.

## Graphical abstract

Prognostic factors in patients with ependymoma (EPN) remain controversial. Based on the SEER database, we constructed prognosis models for EPN 5‐year and 10‐year survival rates. It may provide a reference for clinicians to personalized treatment.

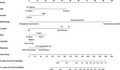

## 1 BACKGROUND

Ependymomas (EPN) is a primary central nervous system tumor, which usually originates from ependymal cells or the central canal of the spinal cord. It is common in the posterior fossa in children and supratentorial and spinal cord in adults.^1^ EPN is common in adolescents and children, with slightly more males than females. EPN account for 5% and 4% of the primary central nervous system tumors in children and adults, according to the central brain tumor registry of the united states (CBTRUS).^2^ According to the histopathological criteria of ependymoma, the World Health Organization (WHO) classified it into three grades: grade I (myxopapillary EPN), grade II (classic EPN), and grade III (anaplastic EPN). At present, surgical operation is still an important component of the standard treatment for ependymoma patients.^3^, ^4^

The prognostic factors of ependymoma are still controversial. Previous studies are often based on the cohort statistical analysis of a small number of people, and the prognosis results are quite different. Even experienced clinicians still have great challenges in predicting the survival time of patients. Moreover, there are differences in medical technology among different medical institutions, which brings greater challenges to the prognosis prediction of ependymoma patients. For neurosurgeons, it is very important to use the clinical data of patients to build an accurate tool to predict the survival probability. Physicians and patients will benefit from a readily available and intuitive predictive model tool that can assess survival outcomes through demographic, histopathology, and surgical approaches in clinical practice.

The nomogram is a common clinical statistical method, which scores the risk factors and then plays a role in predicting the prognosis of the tumor. Unlike previous studies, we included more prognostic factors and a large sample of patients with EPN. The Surveillance, Epidemiology, and End Results (SEER) database is a cancer population registry in the United States that collects basic patient information, clinicopathological characteristics, and treatment‐related data covering nearly one‐third of the U.S. population.^5^ In this study, we screened prognostic risk factor variables for EPN patients for statistical analysis using the SEER database and presented 5‐year and 10‐year survival probabilities using a nomogram.

## 2 METHODS

### 2.1 Study population

**FIGURE 1:**
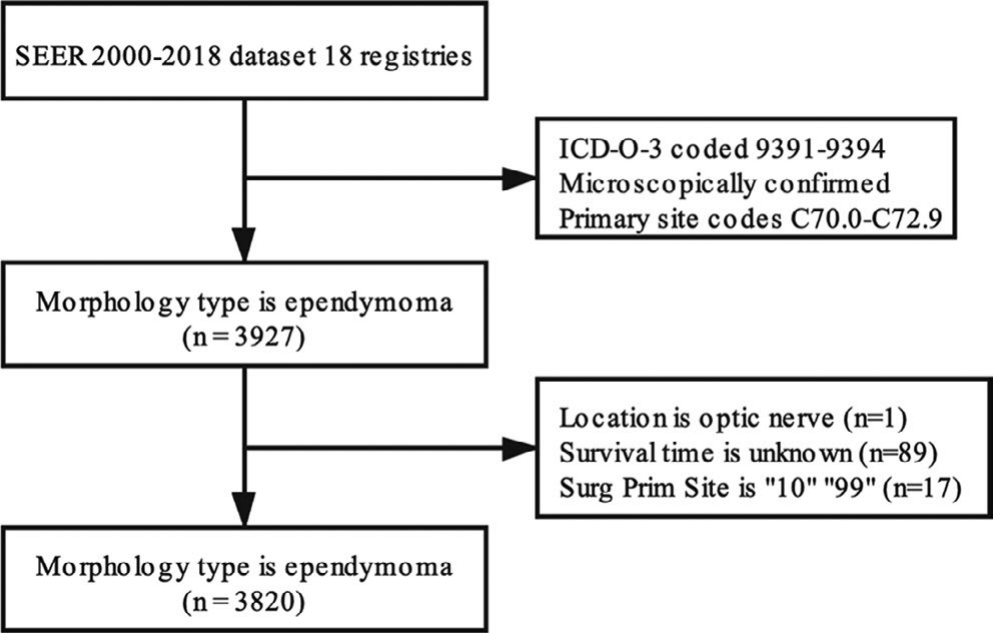
Flowchart of participant inclusion and exclusion

We obtained the complete 2000–2018 dataset online from the Surveillance, Epidemiology, and End Results (SEER) program of the National Cancer Institute (released in Nov 2020). These data sets contain basic patient information from the following 18 registries in the United States (the year of data collection): San Francisco‐Oakland SMSA (2000), Connecticut(2000), Detroit (Metropolitan) (2000), Hawaii(2000), Iowa (2000), New Mexico(2000), Seattle (Puget Sound) (2000), Utah(2000), Atlanta (Metropolitan) (2000), San Jose‐Monterey(2000), Los Angeles(2000), Alaska Natives(2000), Rural Georgia(2000), California excluding SF/SJM/LA(2000), Kentucky(2000), Louisiana(2000), New Jersey(2000), and Greater Georgia(2000). The data of patients with ependymoma, coded 9391–9394 in the International Classification of Diseases for Oncology, third edition (ICD‐O‐3), and confirmed by microscopical diagnosis were screened. And the primary site topography codes were C70.0–C72.9. The flowchart of case inclusion and exclusion criteria is shown in Figure 1.

### 2.2 Study design

SEER demographic data were extracted including patient age, gender, race, morphological diagnosis, location, size, tumor laterality, surgical status, overall survival time, and the survival status at the time and of diagnosis. The ICD‐O‐3 code was used to define the tumor morphological diagnosis: 9391 for Ependymoma, NOS; 9392 for Ependymoma, anaplastic; 9393 and 9394 for Myxopapillary ependymoma, malignant. The primary tumor site was defined by topography codes: supratentorial (ST, C70.0 and C70.2–C71.4), posterior fossa (PF, C71.6–C71.7), spine (SP, C70.1, C72.0–C72.1, and C72.5), and other/unknown (C71.5, C71.8–C71.9, and C72.8–C72.9). Tumor size: 0–9 mm, 10–29 mm, 30–59 mm, 60 mm+, unknown. Tumor laterality: only one side and bilateral side. RX Summ‐‐Surg Prim Site (1998+): No surgery and biopsy only for codes 00 and 20; partial resection for codes 21, 22, 40, and 90; gross total resection for codes 30 and 55. We divided the population into three groups according to age: the children group (0–19 years), the adults’ group (20–49 years), and the elderly group (50+ years). The race includes white, black, and other groups.

### 2.3 Nomogram development and statistical analysis

EPN patients meeting the criteria were randomly divided into the development group and the validation group in a 7:3 ratio (createDatapartition package). Factors selected from least absolute shrinkage and selection operator (LASSO) regression analysis were associated with the prognosis of ependymoma.^6^ A nomogram was developed based on the minimum variable results of LASSO regression for EPN patients by the development group. Concordance index (C‐Index) was used to quantify the discrimination. The “rms,” “foreign,” and “survival” R packages are used to evaluate the consistency of the nomogram model and make the calibration curves. The 5‐year and 10‐year survival rates were the endpoints of the nomogram. In the internal validation, 500 repeated samples were used for bootstrap analysis, and the 5‐year and 10‐year survival benefits were compared by the decision curve analysis (DCA).^7^ The prognosis (PI) was calculated and the optimal cutoff value was determined using the “survivalROC” package and the population was divided into high‐risk and low‐risk groups. Kaplan–Meier (KM) method was used to draw survival curves of patients in the development group and validation group. The “survdiff” package was used for the log‐rank test and *p* < 0.05 was considered to be statistically significant.

R software 4.0.5 (https://www.r‐project.org) was used to construct the nomogram and statistical analysis of all data. All datasets come from the SEER∗Stat software (version 8.3.9, username: 10901‐Nov2020).

## 3 RESULTS

### 3.1 Patient baseline characteristics

Our study included 3820 patients diagnosed with ependymoma from the SEER database between 2000 and 2018. Of all enrolled patients, 2676 (70%) were placed in the development set and 1144 (30%) in the validation set. There were 1947 males (51.0%) and 1,873 females (49.0%). In addition, 1262 cases (33.0%) were older than 50 years old, 1538 cases (40.3%) aged 20–49 years old, and 1020 cases (26.7%) aged 0–19 years old. In the development group, the median follow‐up period was 78 months (range 1–227 months). The 5‐ and 10‐year survival rates were 59.2% and 32.3%, respectively. For the validation group, the median follow‐up period was 80 months (range 1–227 months). The 5‐year and 10‐year survival rates were 58.4% and 31.9%, respectively. The detailed clinical data of the patients are shown in Table 1.

**TABLE 1:** Baseline characteristics of ependymoma patients from the SEER database

Characteristics	All patients N = 3820 (%)	Training set N = 2676 (%)	Validation set N = 1144 (%)	*p* value
Age(years)				0.003
0–19	1020 (26.7)	736 (27.5)	284 (24.8)	
20–49	1538 (40.3)	1067 (39.9)	471 (41.2)	
50+	1262 (33.0)	873 (32.6)	389 (34.0)	
Gender				*p* < 0.001
Male	1947 (51.0)	1369 (51.2)	578 (50.5)	
Female	1873 (49.0)	1307 (48.8)	566 (49.5)	
Race				0.475
White	3150 (82.5)	2199 (82.2)	951 (83.1)	
Black	364 (9.5)	265 (9.9)	99 (8.7)	
Other	306 (8.0)	212 (7.9)	94 (8.2)	
Morphology				*p* < 0.001
9391	3006 (78.7)	2075 (77.5)	931 (81.4)	
9392	722 (18.9)	537 (20.1)	185 (16.2)	
9393–9394	92 (2.4)	64 (2.4)	28 (2.4)	
Location				*p* < 0.001
ST	470 (12.3)	329 (12.3)	141 (12.3)	
PF	824 (21.6)	567 (21.2)	257 (22.5)	
SP	1782 (46.6)	1255 (46.9)	527 (46.1)	
Other/unknown	744 (19.5)	525 (19.6)	219 (19.1)	
Size (mm)				0.040
0–9 mm	75 (2.0)	55 (2.1)	20 (1.7)	
10–29 mm	553 (14.5)	393 (14.7)	160 (14.0)	
30–59 mm	730 (19.1)	493 (18.4)	237 (20.7)	
60 mm+	1148 (30.1)	790 (29.5)	358 (31.3)	
Unknown	1314 (34.4)	945 (35.5)	369 (32.3)	
Laterality				*p* < 0.001
Only one side	525 (13.7)	376 (14.1)	149 (13.0)	
Bilateral side	3295 (86.3)	2300 (85.9)	995 (87.0)	
Resection				*p* < 0.001
No surgery	813 (21.3)	572 (21.4)	241 (21.1)	
Partial resection	1359 (35.6)	946 (35.4)	413 (36.1)	
Gross resection	1648 (43.1)	1158 (43.3)	490 (42.8)	

Abbreviations: 9391, Ependymoma, NOS; 9392, Ependymoma, anaplastic; 9393–9394, Myxopapillary ependymoma, malignant; PF, posterior fossa; SP, spine; ST, supratentorial.

### 3.2 Feature selection and prognostic signature building

**FIGURE 2:**
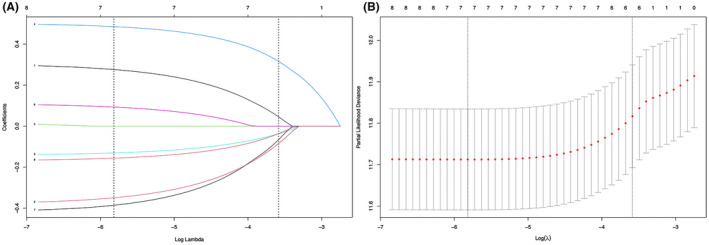
LASSO regression model was used to select characteristic impact factors. A, LASSO coefficients of eight features; B, Selection of tuning parameter (*λ*) for the LASSO model

We reduced the initial eight characteristics of 2676 patients in the development cohort to seven potential predictors of survival: age (coefficient, 0.276),gender (coefficient, −0.349), morphology (coefficient, 0.485),location (coefficient, −0.130),size (coefficient, 0.093),laterality (coefficient, −0.386),and resection (coefficient, −0.156) (Figure 2). LASSO regression model was used to select characteristic variables. Dashed lines are drawn vertically at the optimal value (as used with a minimum value of “min” as the criterion, Figure 2).

### 3.3 Nomogram construction and validation

**FIGURE 3:**
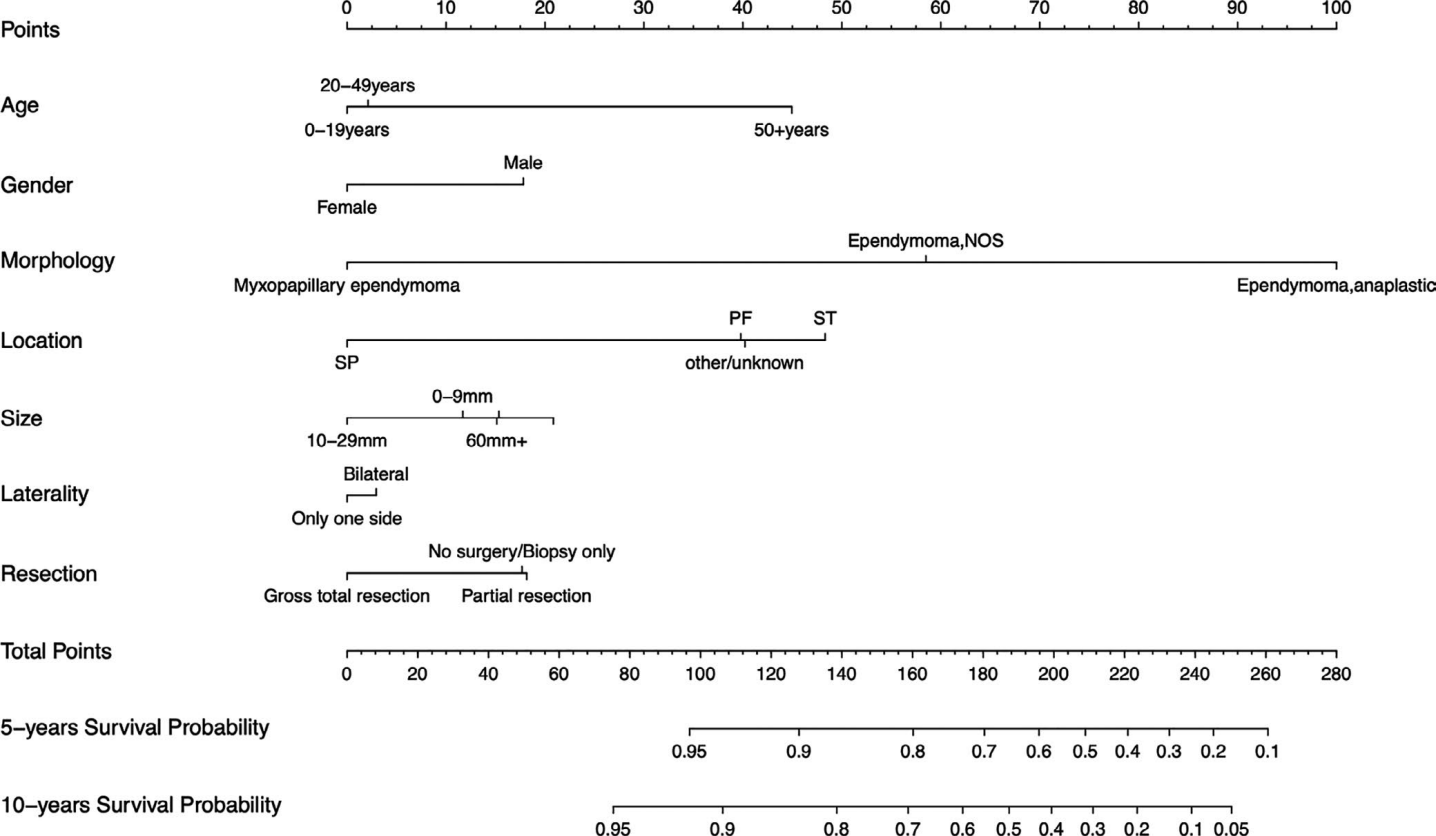
Nomograms predicting 5 ‐ and 10‐year survival rates. ST: supratentorial; PF: posterior fossa; SP: spine

Clinical variables screened by LASSO were collected from the training set, including age, gender, morphology, location, size, laterality, and resection. Using seven variables in the development set, the line diagram of 5‐year and 10‐year survival probability was constructed (Figure 3). The results showed that the correlation between the histological type and prognosis was the strongest, followed by location, age, size, resection, sex, and laterality. The survival probability of a single patient can be calculated simply and intuitively from the score of each selected variable. The scores of variables in the Nomogram are shown in Table 1.

**FIGURE 4:**
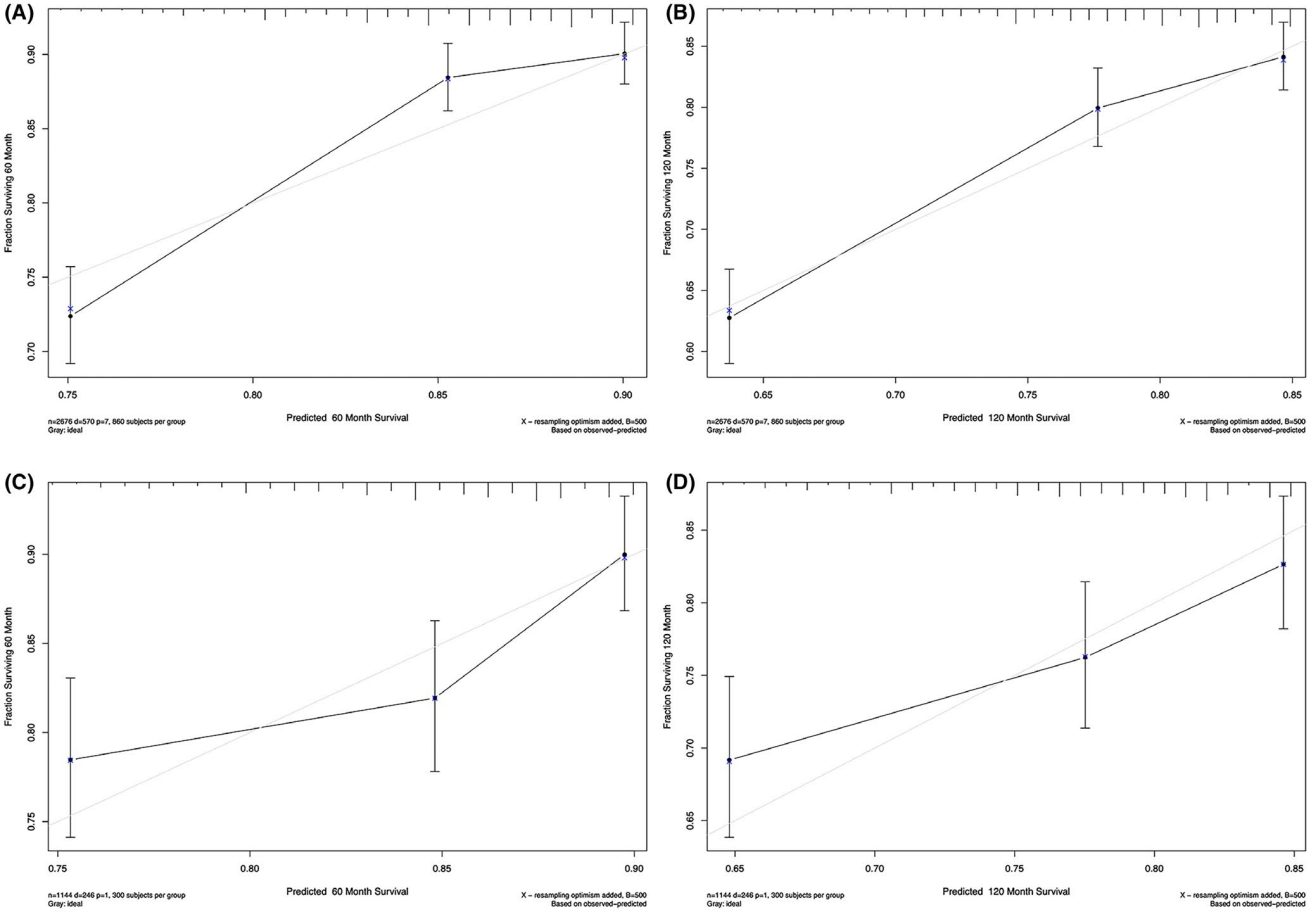
Calibration plot of prognostic nomogram for each cohort. A, 5‐year calibration plot of the development set. B, 10 years calibration plot of the development set; C, 5‐year calibration plot of the validation set; D, 10‐year calibration plot of the validation set

In the development group and validation group, the C‐index of the nomogram prediction model was 0.642 and 0.615, respectively. The actually predicted curve is in good agreement with the verified curve (Figure 4). The model shows good consistency in both the training set and the verification set.

**FIGURE 5:**
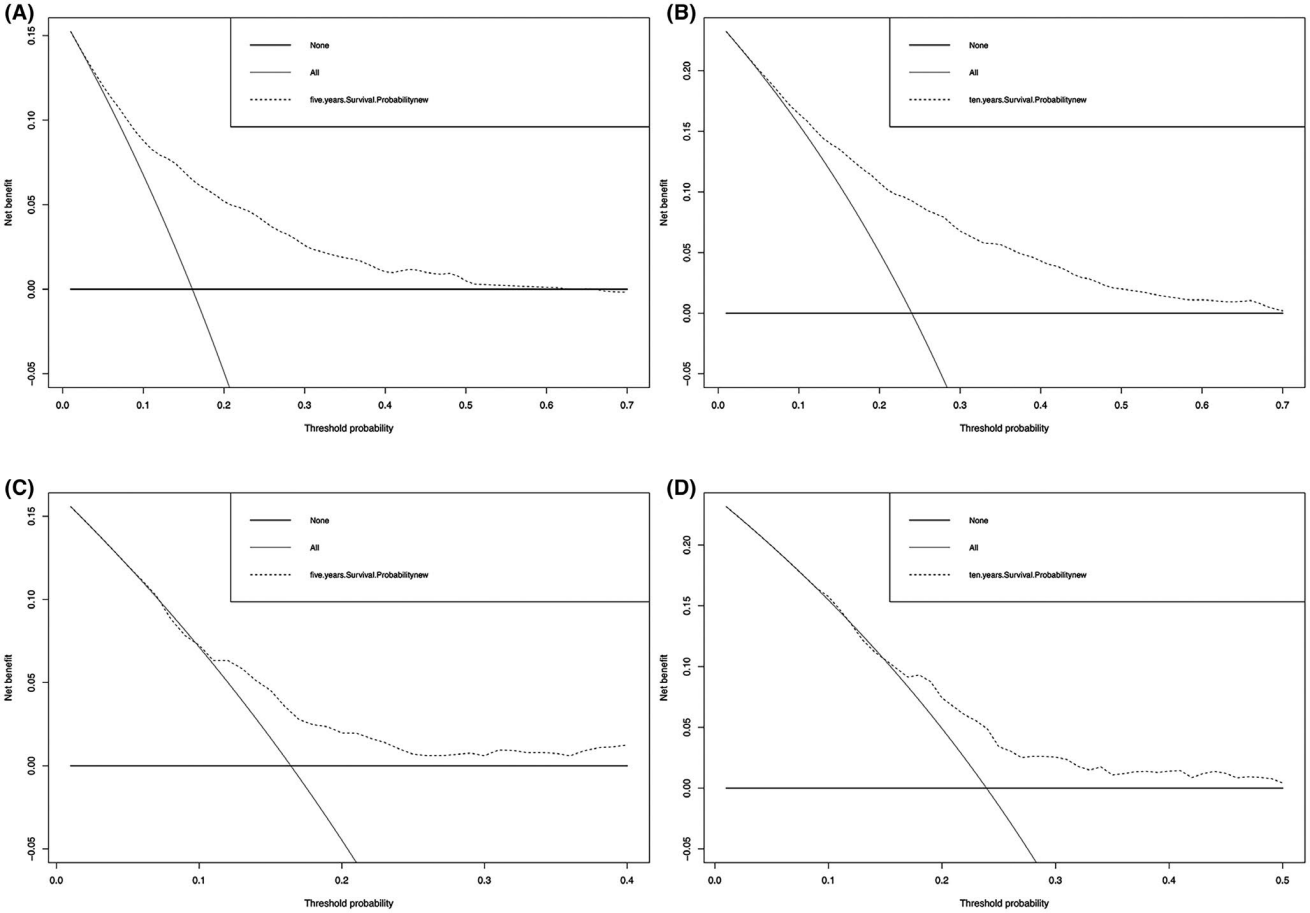
Prognostic decision curve analysis (DCA) of patients with ependymoma. A, Development set 5‐year survival DCA; B, Development set 10 years survival DCA; C, Verify set 5‐year survival DCA; D, Verify set 10‐year survival DCA

After determining the accuracy of the prediction model, we further analyzed it through DCA. The results showed that the histogram had a wide threshold probability range and had good clinical applicability in predicting 5‐year and 10‐year survival rates for ependymoma, with a higher net benefit (Figure 5).

### 3.4 Survival analysis based on PI stratification

**FIGURE 6:**
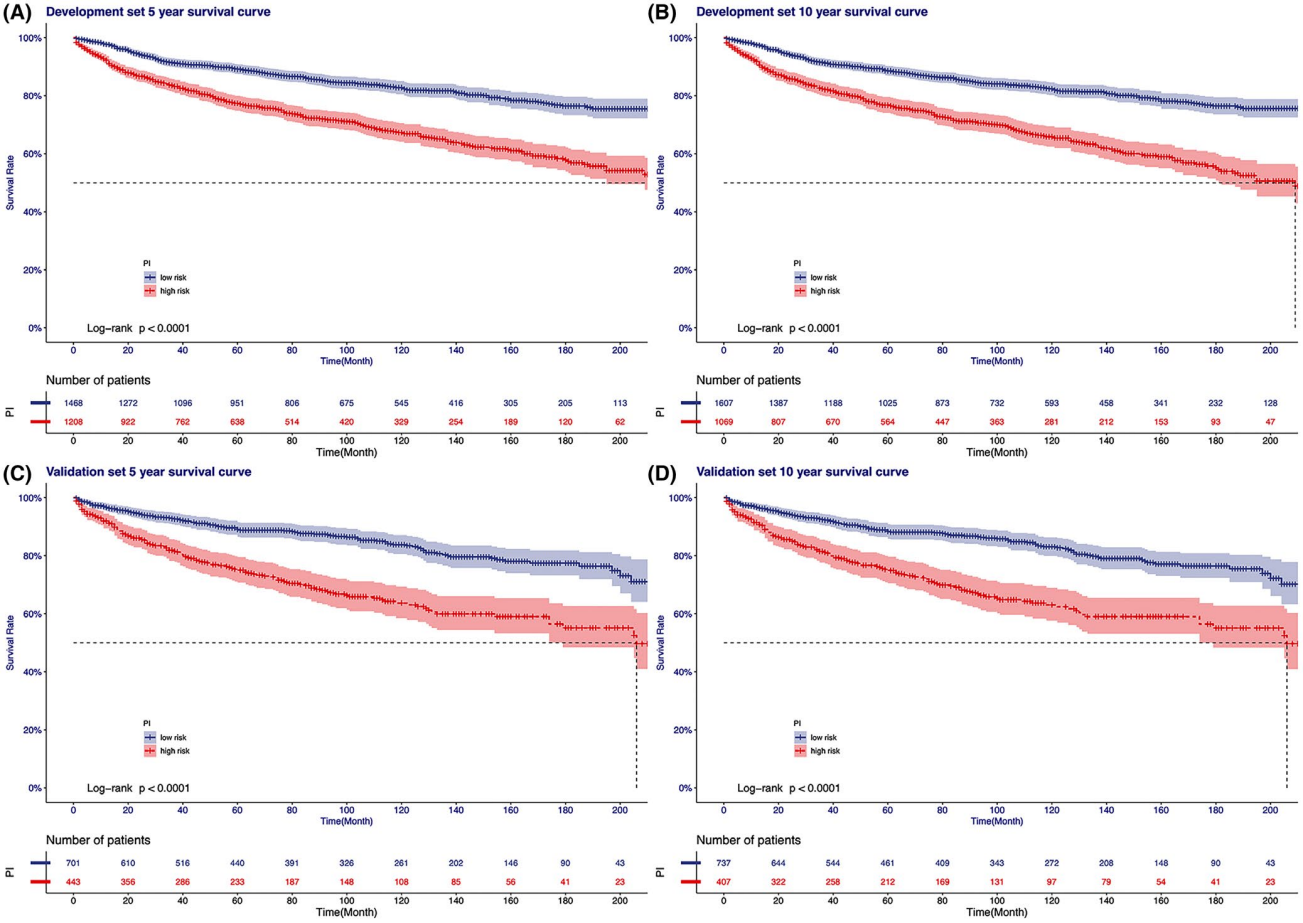
Kaplan–Meier method was used to estimate survival by prognosis index (PI). A, Development set: 5‐year survival curve with cut‐off point; B, Development set: 10‐year survival curve with cut‐off point; C, Validation set: 5‐year survival curve with cut‐off point; D, Validation set: 10‐year survival curve with cut‐off point

In this study, seven variables were used to calculate the PI, and the survival time was used as the cut point to calculate the optimal cut point of PI. The optimal PI cutoff points of the development set and validation set are 5.4 and 6.4. The development validation cohort was divided into high‐risk group and low‐risk group according to different PI cut‐off values. The 5‐year and 10‐year survival curves were drawn and the individual survival number and time data were included. Log‐rank test results showed that there were significant differences between the high‐risk group and the low‐risk group (*p* < 0.0001). (Figure 6).

## 4 DISCUSSION

In this study, we used potential prognostic factors in patients with pathologically diagnosed ependymoma to construct clinical prognostic models of 5‐year and 10‐year probability of survival by obtaining patient data from the SEER database. Our results suggest that age, gender, morphology, location, size, laterality, and resection may be important predictors of survival in patients with ependymoma. Although these clinical features are also prognostic factors for other cancers, their role in the prognosis of patients with ependymomas remains controversial.^8^, ^9^ The prediction model for EPN is rare, and the stratification of clinical data is not detailed enough.^10^, ^11^ There is a lack of a comprehensive prediction model of ependymoma suitable for all ages and tumor locations (brain and spinal cord). To our knowledge, this is the largest retrospective study to stratify the clinical data of patients with ependymoma in more detail and has a wider clinical application than other models.

Our study constructed an integrated predictive model for patients with ependymoma. Consistent with previous reports, we finally found that seven clinical variables were predictors of survival.^11^, ^12^ We found that morphological diagnosis is the strongest factor affecting the prognosis. Deng et al. also found that there were significant differences in the overall survival of ependymoma patients between children and adults.^11^ However, in their study, only grade II (classic EPN) and grade III (anaplastic EPN) were included, and grade I (myxopapillary EPN) was not included. In general, according to the World Health Organization classification, myxopapillary ependymoma (MPE) is considered benign (WHO grade I) and has a good prognosis.^13^ We found that the second major factor affecting the prognosis of patients was age. As we all know, cancer is considered to be an aging disease, and a common risk factor for almost all types of cancer is age, which may be related to age‐related decline in immune function and reduced ability of gene repair.^14^ The third major prognostic factor is tumor location. Therefore, we grouped the tumors according to the anatomical location and found that the prognosis of the tumors in the intracranial EPN (supratentorial and posterior fossa) was worse than that of the spinal cord. Previous reports also show that the different anatomical sites appear to be related to clinical prognosis by analyzing the histological characteristics of 238 patients with ependymoma.^15^ In fact,studies have shown that the biological mechanism of poor prognosis of supratentorial tumors is that the mitosis of tumor cells is relatively active and complex, and it is more difficult to define the tumor boundary and complete surgical resection..^16^

In addition, the correlation between gender, tumor size, and surgical condition has been confirmed by relevant studies.^10^, ^11^ Similarly, our nomogram also confirmed that these factors are related to the prognosis of ependymoma patients. Previous studies have shown that male are an important prognostic factor for poor prognosis of ependymoma, especially in male children.^11^, ^17^ Tumor size is an independent predictor of the prognosis of many solid tumors, and its space‐occupying effect is very important in evaluating the prognosis of cancer patients. In a study of intracranial ependymoma (ICD‐O‐3: C71.0–C71.9), tumor size was found to be an independent prognostic factor in adults.^11^ The difference is that we added patients with tumors in the spinal cord (ICD‐O‐3: C70.1, C72.0–C72.1, and C72.5), and tumor size was still a prognostic factor. In many studies, surgical treatment is considered to be the most important part of the standard treatment for ependymoma patients.^12^ Consistent with our results, the prognosis of total resection is better than that of partial resection.^18^ This will guide us to achieve total tumor resection as far as possible under the premise of not damaging the nerve function, so as to make the prognosis of patients better.

We developed a new nomogram using retrospective clinical cohort data from the SEER clinical database with moderate C‐index and calibration curve results. The results showed that the model constructed by seven prognostic factors obtained from LASSO was relatively stable and reliable.^6^ Our prognostic model has moderate net benefit and is validated by DCA. The abscissa and ordinate of the decision curve are threshold probability and net benefit, respectively, and it is a simple way to evaluate clinical prediction models. Therefore, the nomogram we developed can directly show the 5‐year and 10‐year survival probability to patients, helping clinicians to provide a reference for patients to make decisions about disease treatment.^19^

This study has some limitations. First, we only carried out internal verification of the data, hoping to get external verification in the real world in the future. Second, as it is a retrospective cohort study, potential selection bias is inevitable. Data screening may exclude patients with missing information on the variables collected, which may lead to selection bias and lower C‐index in this study compared with other models. Third, some treatments, such as radiotherapy and chemotherapy, have not been included in the prognosis model, so their differentiation ability is limited. Therefore, further prospective studies are planned to verify the accuracy of the prognosis model.

## 5 CONCLUSIONS

We constructed and internally validated a more broadly applicable nomogram for predicting 5 ‐ and 10‐year survival in patients with EPN. The new nomogram can be used as a simple clinical prediction tool to provide personalized service for patients.

## 6 CONFLICT OF INTEREST

None declared.

## Supporting information

Additional file S1

Additional file S2

Additional file S3

## Data Availability

Retrieved from the SEER* STAT database according to the SEER research data usage protocol.
