# Supplementary material for: Development and validation of prognostic nomogram in ependymoma: A retrospective analysis of the SEER database
Source: Cancer Med. 2021 Aug 3;10(17):6140–8. doi: 10.1002/cam4.4151 (PMC8419756; doi:10.1002/cam4.4151)
Supplement: Supplementary file 1 — Additional file S1 [file CAM4-10-6140-s002.docx]

**Supplementary Table 1. Detailed scores for all variables in nomogram**

| **Variable** | **Nomogram Score** |
| --- | --- |
| Age(years) |  |
| 0-19 | 0 |
| 20-49 | 2 |
| 50+ | 45 |
| Gender |  |
| Male | 18 |
| Female | 0 |
| Morphology |  |
| 9391 | 59 |
| 9392 | 100 |
| 9393 - 9394 | 0 |
| Location |  |
| ST | 48 |
| PF | 40 |
| SP | 0 |
| Other/unknown | 40 |
| Size (mm) |  |
| 0-9mm | 12 |
| 10-29mm | 0 |
| 30-59mm | 15 |
| 60mm+ | 15 |
| Unknown | 21 |
| Laterality |  |
| Only one side | 0 |
| Bilateral side | 3 |
| Therapy |  |
| No surgery | 18 |
| Partial resection | 18 |
| Gross resection | 0 |

9391: Ependymoma, NOS; 9392: Ependymoma, anaplastic; 9393-9394: Myxopapillary ependymoma, malignant; ST: supratentorial; PF: posterior fossa; SP: spine
